# Supplementary material for: Autophagy regulates the maturation of hematopoietic precursors in the embryo
Source: Nat Commun. 2024 Mar 15;15:2255. doi: 10.1038/s41467-024-46453-y (PMC10943005; doi:10.1038/s41467-024-46453-y)
Supplement: Supplementary file 1 — Supplementary Information [file 41467_2024_46453_MOESM1_ESM.pdf]

Supplemental information

Autophagy regulates the maturation of hematopoietic precursors in the embryo

Yumin Liu, Linjuan Shi, Yifan Chen, Sifan Luo, Yuehang Chen, Hongtian Chen, Wenlang Lan, Xun Lu, Zhan Cao, Zehua Ye, Jinping Li, Bo Yu, Elaine Dzierzak, and Zhuan Li

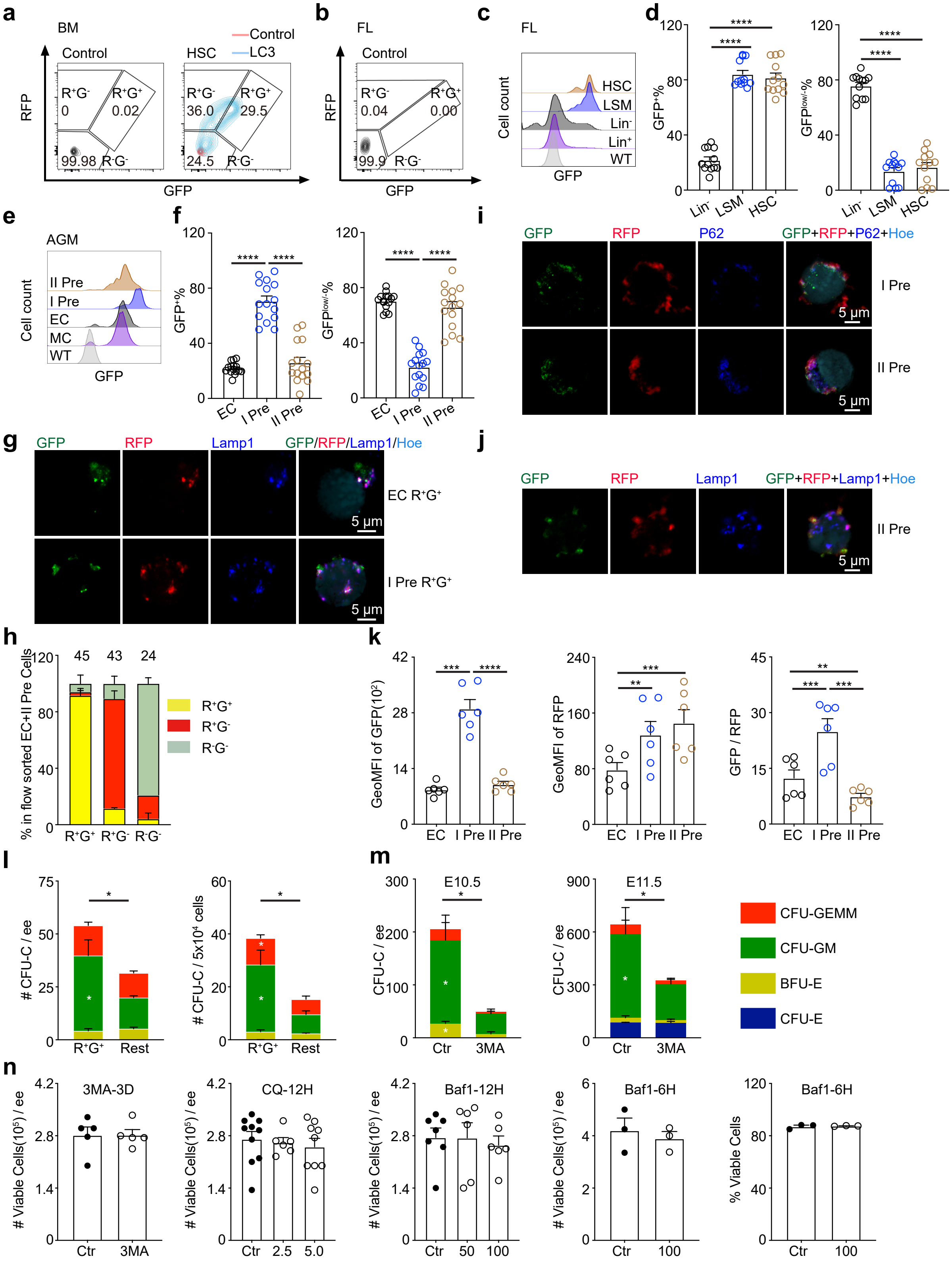
a
BM
Control
HSC
Control
LC3
RFP
GFP
R+G- 0
R+G+ 0.02
99.98 R-G-
R+G- 36.0
R+G+ 29.5
24.5 R-G-
b
FL
Control
R+G- 0.04
R+G+ 0.00
99.9 R-G-
c
FL
Cell count
HSC
LSM
Lin-
Lin+
WT
d
GFP+%
GFPlow/-%
Lin-
LSM
HSC
e
AGM
II Pre
I Pre
EC
MC
WT
f
EC
I Pre
II Pre
i
GFP
RFP
P62
GFP+RFP+P62+Hoe
5 μm
g
Lamp1
GFP/RFP/Lamp1/Hoe
EC R+G+
I Pre R+G+
j
GFP+RFP+Lamp1+Hoe
h
% in flow sorted EC+II Pre Cells
45
43
24
R+G+
R+G-
R-G-
k
GeoMFI of GFP(10²)
GeoMFI of RFP
GFP / RFP
l
CFU-C / ee
CFU-C / 5x10⁴ cells
Rest
m
E10.5
E11.5
CFU-C / ee
Ctr
3MA
CFU-GEMM
CFU-GM
BFU-E
CFU-E
n
3MA-3D
CQ-12H
Baf1-12H
Baf1-6H
Viable Cells(10⁵) / ee
% Viable Cells
2.5
5.0
50
100

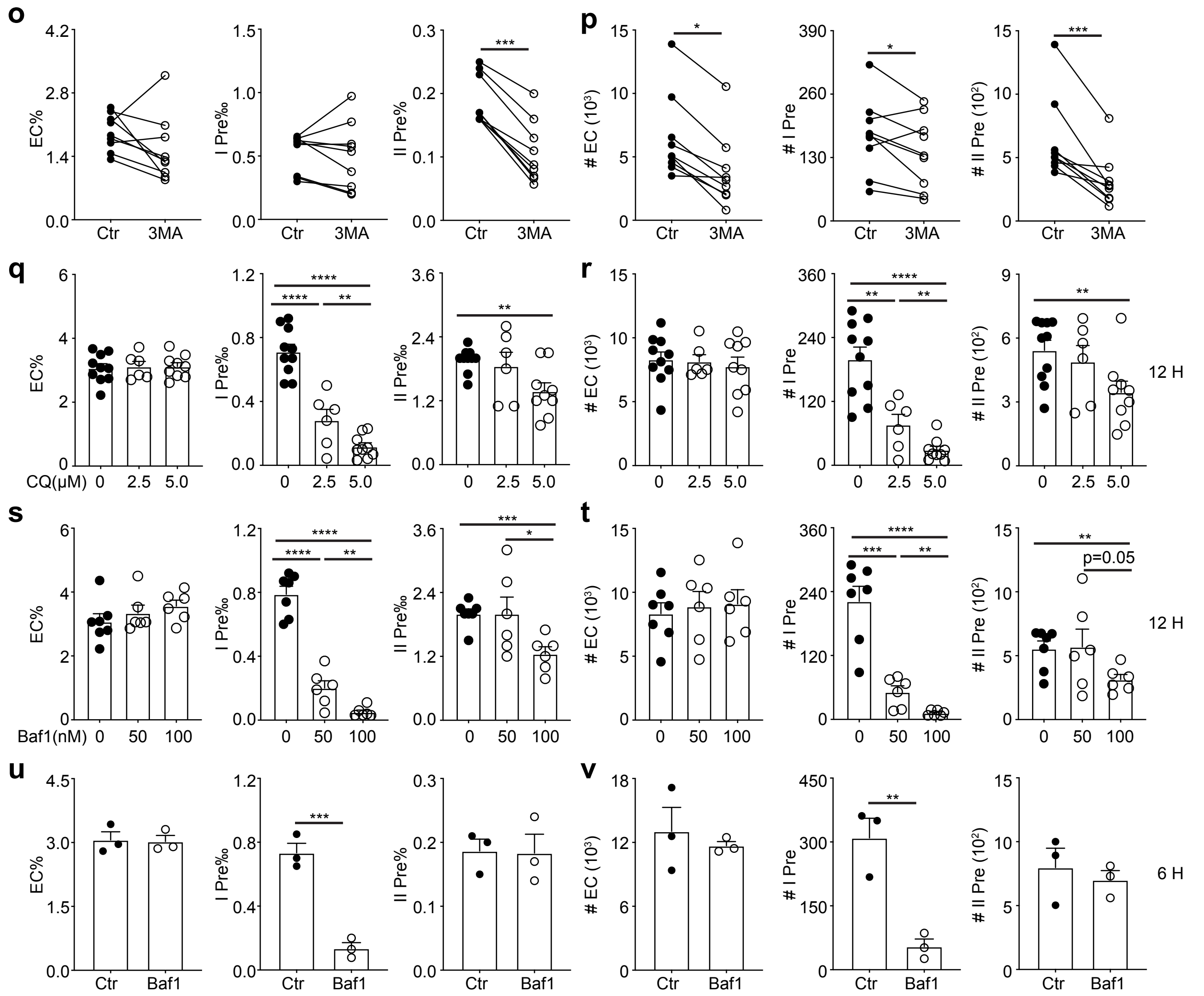

Figure S1. In vitro inhibition autophagy results in the reduction of pre-HSCs and HPCs.

a Flow cytometric analysis showing the RFP and GFP fluorescence level in Lin$^-$ cKit$^+$Sca1$^+$CD150$^+$CD48$^-$(HSC) cells of adult bone marrow. Wild type (WT) viable cells as a negative control. Red line=WT control, blue=LC3-RFP-EGFP(LC3$^{R/G}$). **b** The gating strategy of RFP and GFP in E12.5 wild type (WT) fetal liver. **c** Flow cytometric analysis showing the GFP fluorescence level in Lin$^+$, Lin$^-$, Lin$^-$Sca1$^+$Mac1^{low} (LSM) and CD201$^+$LSM (HSC) cells of E12.5 LC3$^{R/G}$ fetal liver. WT viable cells as negative control. **d** The percentage of GFP$^+$ and GFP$^{low/-}$ in distinct cell fractions (Lin$^-$, LSM and HSC) of fetal liver. Error bars represent mean ± SEM. n=12

biologically independent embryos, ****$p<0.0001$. **e** Histogram displaying the GFP fluorescence level in WT viable cells and endothelial cells (EC, $CD31^+CD41^-CD45^-$), pre-HSC I (I Pre, $CD31^+CD41^{low}CD45^-$) and pre-HSC II (II Pre, $CD31^+CD45^+$) of E11.5 $LC3^{R/G}$ AGM region. WT viable cells as negative control. **f** The percentage of GFP^+ and $GFP^{low/-}$ cells in distinct cell fractions of $LC3^{R/G}$ AGM region. Error bars represent mean ± SEM. n=15 biologically independent embryos, ****$p<0.0001$. **g** Representative immunostaining data showing the signals of GFP, RFP and Lamp1 in the RFP^+GFP^+ fractions of EC and pre-HSC I cells. GFP=Green, RFP=Red, Lamp1=Blue and Hoechst=light blue. Scale bar=5 μm. **h** The percentages of RFP^+GFP^+, RFP^+GFP^- and RFP^-GFP^- EC and pre-HSC II were confirmed by fluorescence microscopy in the flow-sorted RFP^+GFP^+, RFP^+GFP^- and RFP^-GFP^- ECs and pre-HSC IIs. The total cell number of ECs and pre-HSC II are indicated in the figures. **i** Representative immunostaining showing GFP, RFP and p62 were colocalization in the pre-HSC I and pre-HSC II. GFP=Green, RFP=Red, P62=blue, and Hoechst=light blue. Scale bar=5 μm. **j** Representative immunostaining showing Lamp1 was co-localized with RFP, but not with GFP in the pre-HSC II. GFP=Green, RFP=Red, P62=blue, and Hoechst=light blue. Scale bar=5 μm. **k** Geomean fluorescence intensity (GeoMFI) of GFP, RFP and the GeoMFI ratios of GFP/RFP in the endothelial cells (EC, $CD31^+CD41^-CD45^-$), pre-HSC I (I Pre, $CD31^+CD41^{low}CD45^-$), pre-HSC II (II Pre, $CD31^+CD45^+$) of E11.5 $LC3^{R/G}$ AGM region. Error bars represent mean ± SEM. n=6 biologically independent embryos, **$p<0.01$, ***$p<0.001$, ****$p<0.0001$. **l** Methylcellulose culture data showing the number of CFU-Cs and number of each hematopoietic colony type (indicated by colour bars) from RFP^+GFP^+ and $RFP^{+/-}GFP^-$ cells of E11.5 $LC3^{R/G}$ AGM per embryo equivalent (ee). Error bars represent mean ± SEM. n=3 biologically independent embryos, *$p<0.05$. **m** Methylcellulose culture data showing the number of CFU-Cs and number of each hematopoietic colony type (indicated by colour bars) in E10.5-E11.5 AGM explant culture with or without 3-MA per embryo equivalent (ee). Error bars

represent mean ± SEM. n=3 biologically independent experiments in E10.5 and E11.5 AGM respectively, *p<0.05. **n** The cell number or percentage of viable cells in E10.5-E11.5 AGM explant culture with or without 3-MA, Chloroquine, Bafilomycin A1. Error bars represent mean ± SEM. n⩾3. Chloroquine=CQ. Bafilomycin A1=Baf1. **o** Flow cytometric analysis showing the percentages of EC, pre-HSC I (I Pre), and pre-HSC II (II Pre) in E10.5-E11.5 AGM explant culture with or without 3-MA. Error bars represent mean ± SEM. n=9 biologically independent embryos, ***p=0.0005. **p** The reduced cell numbers of of EC, pre-HSC I (I Pre) and pre-HSC II (II Pre) in E11.5 AGM explant culture with or without 3-MA. Error bars represent mean ± SEM. n=9 biologically independent embryos, *p<0.05, ***p=0.0007. **q-r** Chloroquine reduced the percentages and cell numbers of pre-HSC I (I Pre) and pre-HSC II (II Pre), but not endothelial cells (EC) in E10.5 AGM explant culture for 12-hour culture. Error bars represent mean ± SEM. n⩾6, **p<0.05, ****p<0.0001. Chloroquine=CQ. **s-v** The alteration of the percentage and cell number in endothelial cells (EC), pre-HSC I (I Pre) and pre-HSC II (II Pre) of E10.5-11.5 AGM explant culture with or without bafilomycin A1 for 12 or 6-hour cultures. Error bars represent mean ± SEM. n≥3, *p=0.0259, **p<0.01, ***p<0.001, ****p<0.0001. Bafilomycin A1=Baf1. Statistical significance was determined by one side Student's t-test.

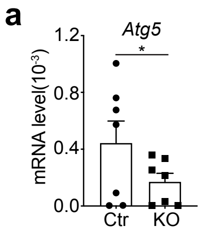

Figure S2. The deletion efficiency of Atg5 in the AGM region. a QRT-PCR data showing the deletion of ATG5 in the AGM endothelial cells. Error bars represent mean ± SEM. n=7 biologically independent embryos, *p=0.0251. Statistical significance was determined by one side Student's t-test.

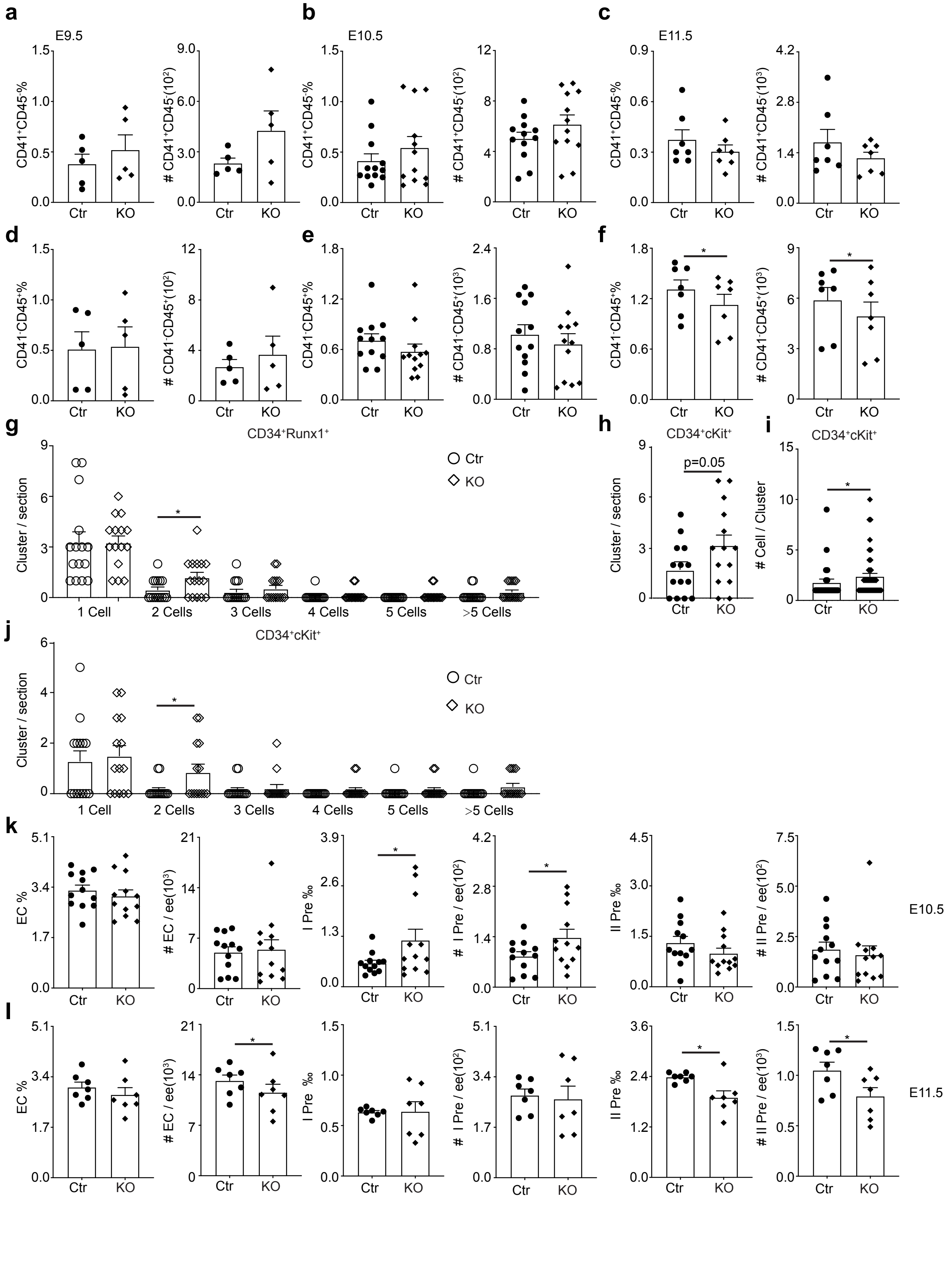
a
E9.5
CD41+CD45-%
CD41+CD45-(10²)
b
E10.5
c
E11.5
CD41+CD45-(10³)
d
CD41-CD45+%
CD41-CD45+(10²)
e
CD41-CD45+(10³)
f
g
CD34+Runx1+
Cluster / section
Ctr
KO
1 Cell
2 Cells
3 Cells
4 Cells
5 Cells
>5 Cells
h
CD34+cKit+
p=0.05
i
Cell / Cluster
j
k
EC %
EC / ee(10³)
I Pre ‰
I Pre / ee(10²)
II Pre ‰
II Pre / ee(10²)
E10.5
l
II Pre / ee(10³)
E11.5

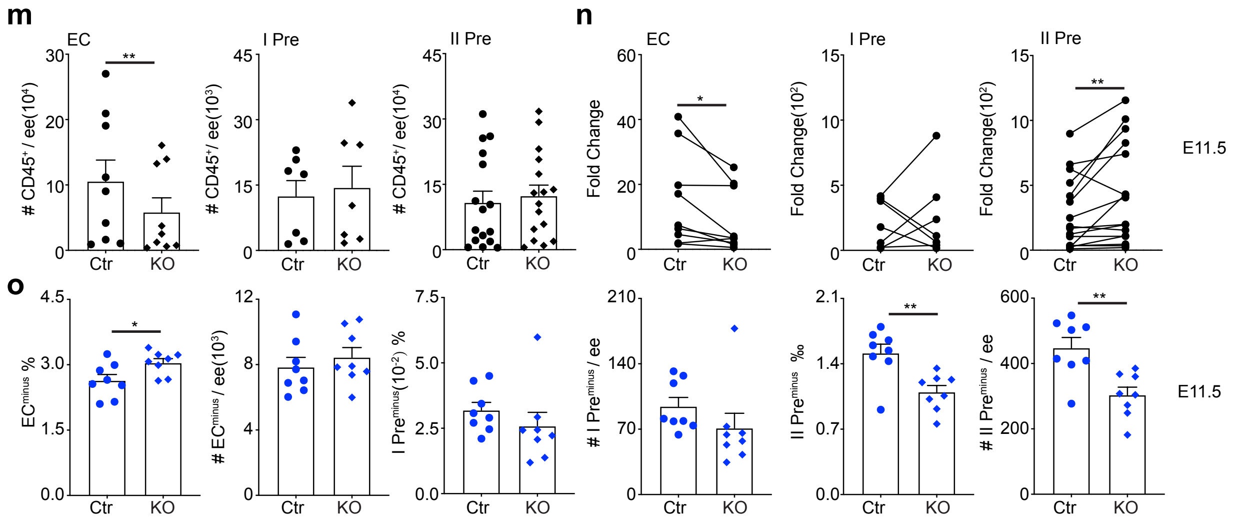

Figure S3. Atg5 deficiency results in the alteration of hematopoietic precursor cell development in the AGM region. a-f The percentages and absolute numbers of CD41^{+}CD45^{-} cells or CD41^{-}CD45^{+} cells in E9.5-E11.5 AGM region by flow cytometric analysis. Error bars represent mean ± SEM. n=5, 12 and 7 biologically independent experiments in E9.5, E10.5 and E11.5 AGM respectively, *p<0.05. **g** Quantification of CD34^{+}Runx1^{+} hematopoietic cluster cells using confocal images. Bars and symbols represent the number of clusters in each category of cluster cell size. Total 30 sections were analysed. Error bars represent mean ± SEM. n=3 biologically independent embryos, *p=0.0275. Statistical significance was determined by Mann-Whitney U-test. **h** The average number of cKit^{+}CD34^{+} hematopoietic clusters in the detected sections. Error bars represent mean ± SEM. n=3 biologically independent embryos. Statistical significance was determined by Mann-Whitney U-test. **i** The average cell number in the detected cKit^{+}CD34^{+} hematopoietic clusters. Error bars represent mean ± SEM. n=3

biologically independent embryos, *p=0.0279. Statistical significance was determined by Mann-Whitney U-test. **j** Quantification of cKit$^+$CD34$^+$ hematopoietic cluster cells by using confocal images. Bars and symbols represent the number of clusters in each category of cluster cell size (total 28 sections were analyzed). Error bars represent mean ± SEM. n=3 biologically independent embryos, *p=0.0344. Statistical significance was determined by Mann-Whitney U-test. **k-l** The percentages and the cell numbers of EC (CD31$^+$CD41$^-$CD45$^-$), pre-HSC I (I Pre, CD31$^+$CD41^{low}CD45$^-$) and pre-HSC II (II Pre, CD31$^+$CD45$^+$) in the E10.5 (k) and E11.5 (l) AGM region. Error bars represent mean ± SEM. n=12 and 7 biologically independent experiments in E10.5 and E11.5 AGM respectively, *p<0.05. **m-n** The number of CD45$^+$ cells (m) and the fold change (the ratio between CD45$^+$ cell number and input cell number) (n) from E11.5 EC, pre-HSC I and pre-HSC II cocultured with OP9-DL1 for 7 days. Error bars represent mean ± SEM. n=9, 7 and 16 biologically independent experiments in EC, I Pre and II Pre respectively, *p=0.0168. **p<0.01. **o** The percentage and the number of EC^{minus}, pre-HSC I^{minus} and pre-HSC II^{minus} in the E11.5 KO AGM region compared with control group. EC^{minus}=EC-EC^{plus}, Pre-HSC I^{minus}=Pre-HSC I-pre-HSC I^{plus}, Pre-HSC II^{minus}=Pre-HSC II-pre-HSC II^{plus}. Error bars represent mean ± SEM. n=8 biologically independent experiments, *p=0.0208, **p<0.01. Statistical significance was determined by one side Student's t-test unless the statistical test was indicated.

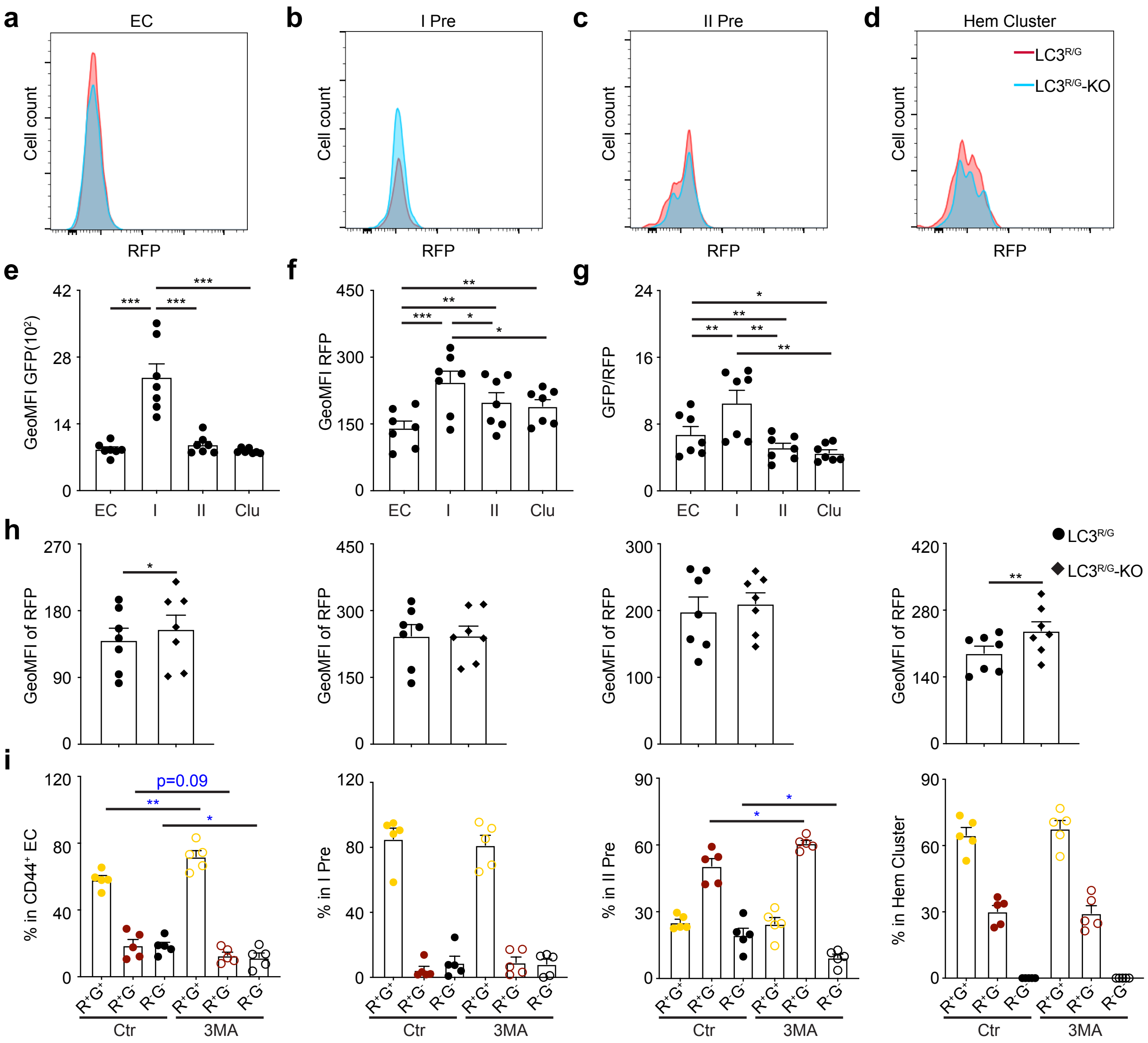
a
EC
b
I Pre
c
II Pre
d
Hem Cluster
LC3R/G
LC3R/G-KO
Cell count
RFP
e
f
g
GeoMFI GFP(10²)
GeoMFI RFP
GFP/RFP
EC
I
II
Clu
h
GeoMFI of RFP
i
p=0.09
% in CD44+ EC
% in I Pre
% in II Pre
% in Hem Cluster
Ctr
3MA

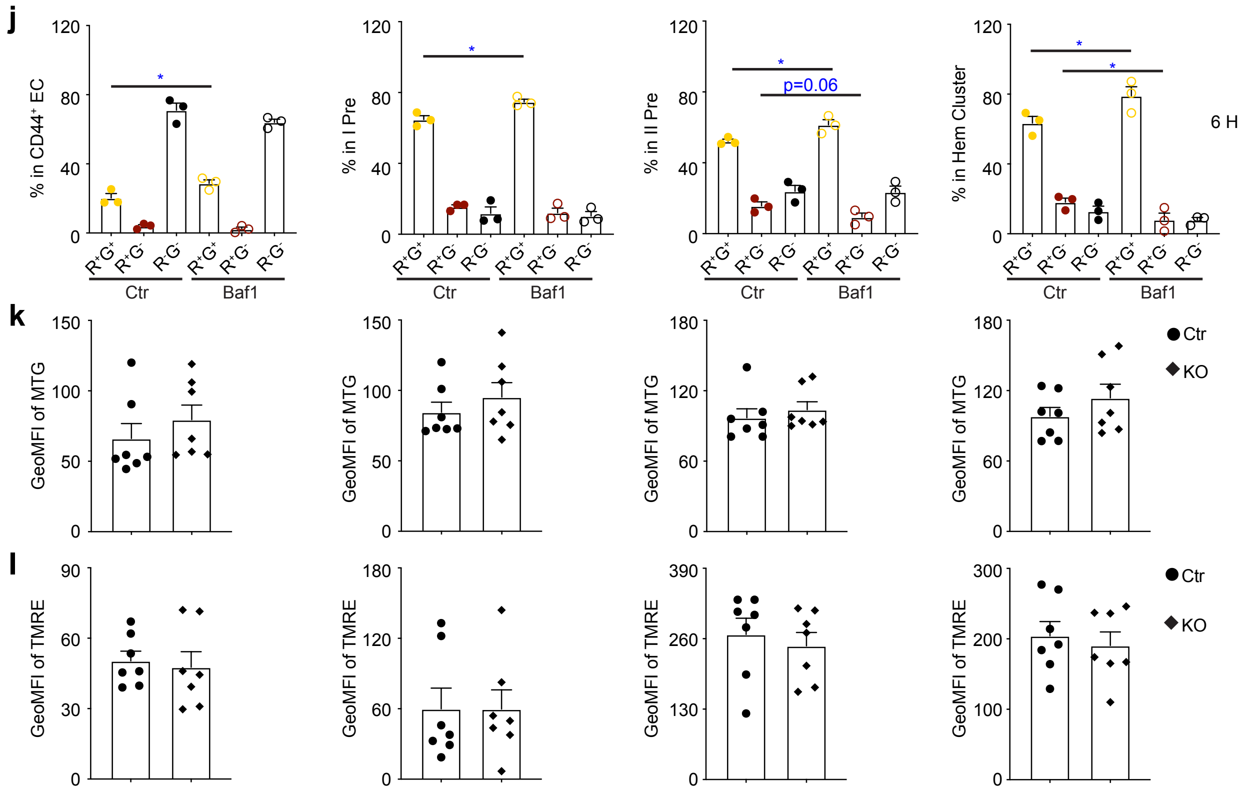

Figure S4. Atg5 deletion impairs the formation of autolysosome and mitochondrial function. a-d Representative histogram showing RFP fluorescence level in the EC ($CD31^{+}CD41^{-}CD45^{-}$), pre-HSC I (I Pre, $CD31^{+}CD41^{low}CD45^{-}$), pre-HSC II (II Pre, $CD31^{+}CD45^{+}$) and hematopoietic clusters (Hem Cluster, $CD31^{+}cKit^{+}$). Red line=$LC3^{R/G}$ group, Blue line=$LC3^{R/G}$-KO group. **e-g** GeoMFI of GFP and RFP signals and the GeoMFI ratios of GFP/RFP in the EC, pre-HSC I, pre-HSC II and hematopoietic clusters of $LC3^{R/G}$. Error bars represent mean ± SEM. n=7 biologically independent embryos, *p<0.05, **p<0.01, ***p<0.001, ****p<0.0001. **h** Comparison analysis of the GeoMFI of RFP signals in the EC, pre-HSC I, pre-HSC II and hematopoietic clusters of $LC3^{R/G}$ and $LC3^{R/G}$-KO. Error bars represent mean ± SEM. n=7 biologically independent embryos, *p=0.0226, **p=0.0041. **i** Comparative analyzing the percentages of $RFP^{+}GFP^{+}$, $RFP^{+}GFP^{-}$ and $RFP^{-}GFP^{-}$ in the EC, pre-HSC I, pre-HSC II and hematopoietic clusters after 3-MA treatment. Error bars represent mean ± SEM. n=5 biologically independent

embryos, $^*p<0.05$, $^{**}p=0.0068$. **j** The percentages of RFP^+GFP^+, RFP^+GFP^- and RFP^-GFP^- in the $CD44^+$EC, pre-HSC I，pre-HSC II and hematopoietic clusters after 6-hour Baf1 treatment. Error bars represent mean ± SEM. n=3 biologically independent embryos, $^*p<0.05$. **k-l** The GeoMFI of MTG or TMRE, in the EC, pre-HSC I，pre-HSC II，and hematopoietic clusters in the control and KO AGM region. Error bars represent mean ± SEM. n=7 biologically independent embryos. Statistical significance was determined by one side Student’s t-test.

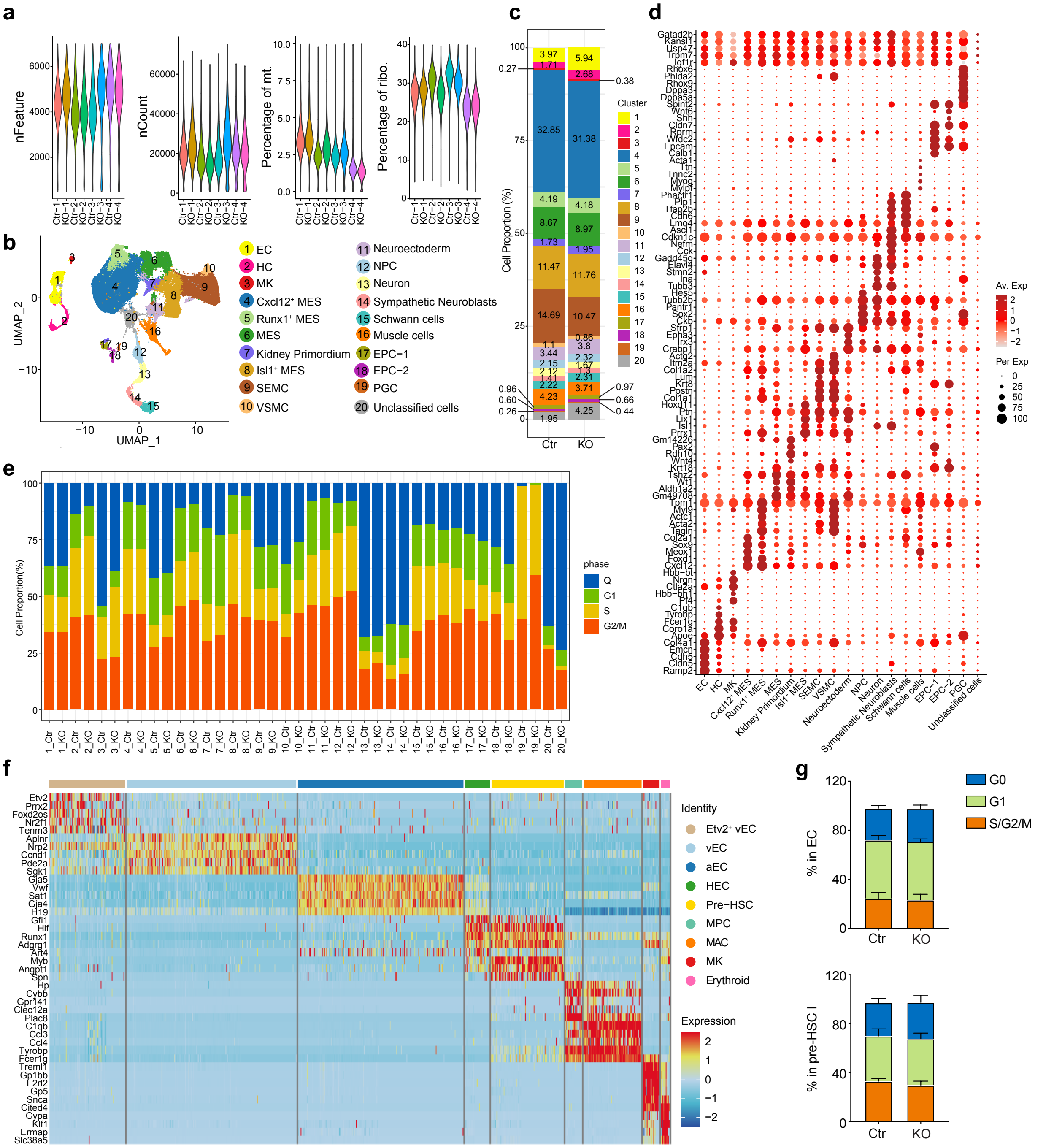

Figure S5. Transcriptomic atlas of the control and Atg5 deficient AGM cells. a Quality control by nFeature, nCount and percentage of mitochondrial and ribosome. mt.=mitchondrial, ribo.=ribosome. **b** UMAP plots visualized the 20 clusters from E10.0 AGM cells. **c** Bar graph

showing the portions of each cluster in the control and KO AGM regions. **d** Dot plots showing the expression level of top 5 significantly differentially expressed genes in each cluster. **e** Bar charts showing the constitutions of cells with different cell cycle phases in the control and KO cells in the scRNA-seq data. **f** Heatmap showing the featured genes of sub-clusters from EC, HC and Mk clusters. **g** Flow cytometric analysis confirmed the cell cycle status of EC and pre-HSC I in the E11.5 AGM region. Error bars represent mean ± SEM. n=4 biologically independent embryos. Statistical significance was determined by one side Student's t-test.

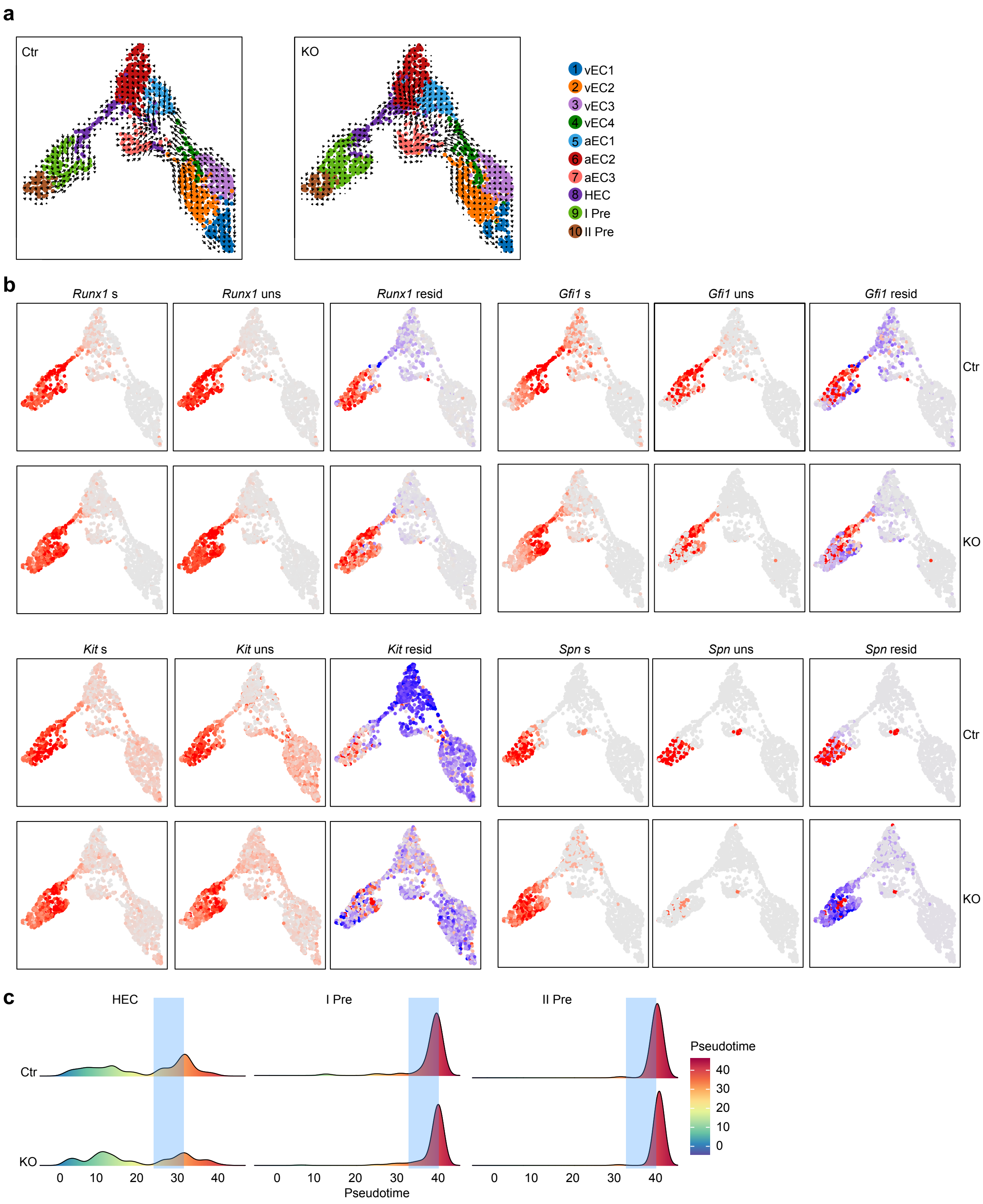

Figure S6. RNA velocito analysis in the process of endothelial to hematopoietic transition.

a The developmental cell dynamics by RNA velocity analysis in the KO and control EC and pre-

HSCs. **b** Velocity estimation for gene expression during EHT, including *Runx1*, *Gfi1*, *Kit* and *Spn* in the KO and control AGM regions. **c** The components of cell (HEC, pre-HSC I and pre-HSC II) in the indicated pseudotime by trajectory analysis. Blue shadows indicate the key part of changes.

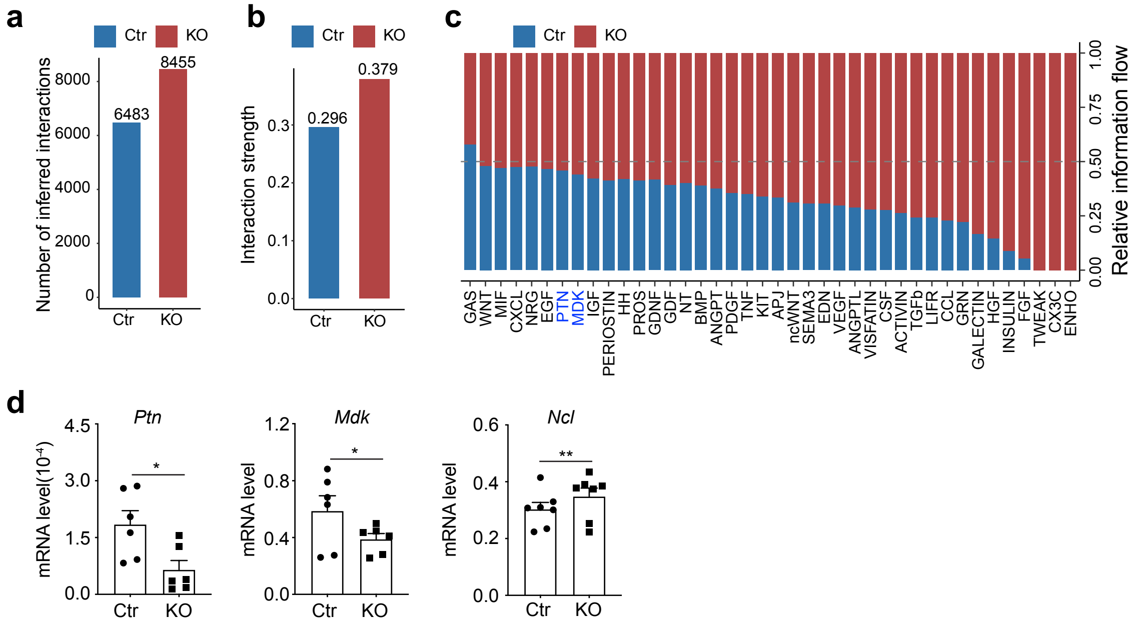

Figure S7. The alteration of interaction between distinct cells in the E10.0 control and Atg5 depleted AGM cells by cell chat analysis. a-b Comparison analysis of the interaction number (a) and strength(b) in all cells of the control and KO group based on the ligand-receptor interactions. **c** The enriched pathways in all cells of E10.0 control and KO AGM regions. **d** QRT-PCR data showing the gene expression of Nclpathways (*Mdk*, *Ptn* and *Ncl*) in the E10.5-E11.0 KO and control endothelial cells. *Ptn=pleiotrophin*, *Mdk=midkine*, *Ncl=nucleolin*. Error bars represent mean ± SEM. n=6, 6 and 7 biologically independent experiments for testing *Ptn*, *Mdk* and *Ncl*, respectively n≥6, *p<0.05, **p=0.0044. Statistical significance was determined by one side Student's t-test.

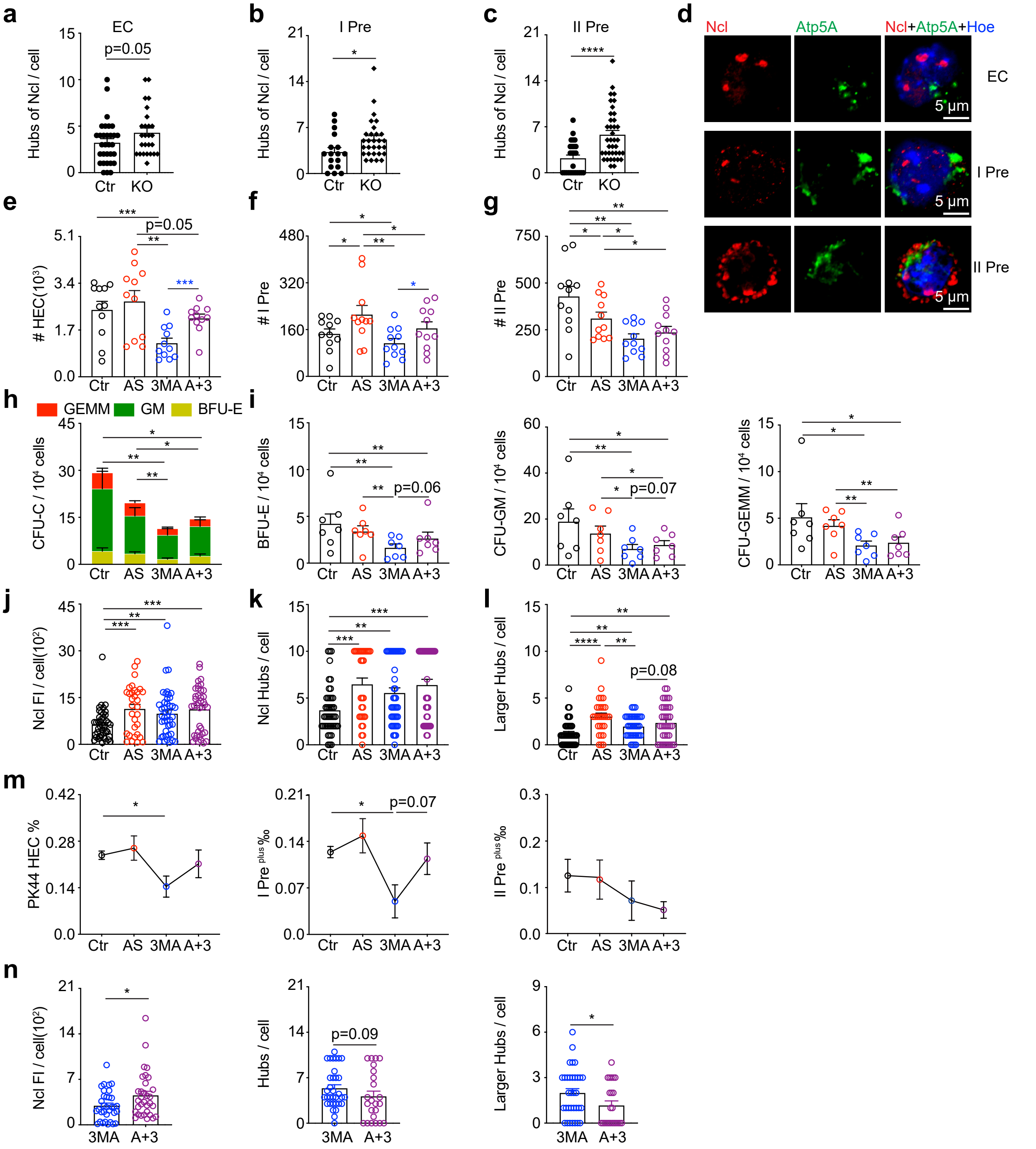

Figure S8. Atg5 regulates endothelial to hematopoietic transition through Ncl pathway. a-c Hubs of Ncl signal in the EC, pre-HSC I (I Pre) and pre-HSC II (II Pre) cells. Error bars represent mean ± SEM. n≥26 cells in EC, n⩾17 in pre-HSC I, n⩾26 in pre-HSC II from 3 biologically

independent experiments. p*=0.0196, p****<0.0001. **d** Representative immunostaining showing rare colocalization between NCL and Atp5A in the EC, pre-HSC I and pre-HSC II. Ncl=Red, Atp5A=Green and Hoechst=Blue. **e-g** E10.5 AGM Explant cultures showing the rescue function in the cell number of HEC and pre-HSC I population after 3-MA and AS1411 treatment, but not in the pre-HSC II. AS=AS1411, the aptamer of Ncl (also known as AGRO100). 3MA=3-MA, the inhibitor of autophagy. A+3=AS1411+3MA. Error bars represent mean ± SEM. n=11 biologically independent embryos, *p<0.05, **p<0.01, ***p=0.0006. **h-i** The total number of CFU-C, BFU-E, CFU-GM and CFU-GEMM per 10^4 AGM cells in the existence of AS1411 and 3-MA after 3 days explant culture. Error bars represent mean ± SEM. n=7 biologically independent embryos, *p<0.05, **p<0.01. **j** Fluorescence intensity (FI) of Ncl in HEC fraction was calculated by image J. Error bars represent mean ± SEM. n⩾31 cells from biologically independent embryos, **p=0.0049, p***<0.001. **k-l** Hubs and larger hubs (maximum length of each hub over one micrometer) of Ncl signal in the HECs. Error bars represent mean ± SEM. n ⩾ 29 cells cells from biologically independent embryos, **p<0.01, ***p<0.001, ****p<0.0001. **m** The percentage of PK44 ($CD31^+$ $CD201^+cKit^+CD44^+CD45^-CD41^-$) and pre-HSC I^{plus} was rescued by AS1411 in the inhibition of 3-MA, but not in pre-HSC II^{plus}. Error bars represent mean ± SEM. n=3 biologically independent embryos, *p<0.05. **n** The Fluorescence intensity of NCL and bigger hubs were rescued by AS1411 after 1 day 3-MA treatment. Error bars represent mean ± SEM. n⩾23 from biologically independent embryos, *p<0.05. Statistical significance was determined by one side Student's t-test.

Table S1. Sample information for 10x genomics sequencing

Genotype	Stage(sp)[a]	No. of embryos(ee)[b]	Cell sorting	Cell number (10^4)	Experiments (n)
Ctr	33-35	4	$Ter119^-$	3.0762	2
KO	33-34	4	$Ter119^-$	1.9986	2
Ctr	33-34	2	$Ter119^-/CD31^+/CD41^+/CD45^{+c}$	1.5941	1
KO	33	2	$Ter119^-/CD31^+/CD41^+/CD45^{+c}$	1.9742	1

a. sp: somite pairs

b. ee: embryo equivalent.

c. adding negative cells up to $3x10^4$ cells.

Table S2. Antibodies used for FACS analysis.

Antibodies; clone	Source	Identifier	Dilutions
CD31-PE; clone MEC13.3	Biolegend	Cat#: 102508; RRID: AB_312915	1:400
CD31-APC; clone MEC13.3	Biolegend	Cat#: 102510; RRID: AB_312917	1:400
CD31-Alexa Flour 700; clone 390	Biolegend	Cat#: 102444; RRID: AB_2832289	1:400
CD41-FITC; clone MWReg30	BD Biosciences	Cat#: 553848; RRID: AB_395085	1:400
CD41-BV510; clone MWReg30	Biolegend	Cat#: 133923; RRID: AB_2564013	1:400
CD41-PE-Cyanine7; clone MWReg30	Biolegend	Cat#: 133916; RRID: AB_11124102	1:400
CD45-PE; clone 30-F11	BD Biosciences	Cat#: 553081; RRID: AB_394611	1:400
CD45-APC; clone 30-F11	Biolegend	Cat#: 103112; RRID: AB_312977	1:400
CD43-BV421; clone S7	BD Biosciences	Cat#: 562958; RRID: AB_2665409	1:400
CD45-FITC; clone 30-F11	BD Biosciences	Cat#: 553079; RRID: AB_394609	1:400
CD44-APC-Cy7; clone IM7	Biolegend	Cat#: 103028; RRID: AB_830785	1:400
CD44-BV605; clone IM7	BD Biosciences	Cat#: 563058; RRID: AB_2737979	1:400
CD45.1-APC; clone A20	BD Biosciences	Cat#: 558701; RRID: AB_1645214	1:400
CD45.2-PE; clone 104	BD Biosciences	Cat#: 560695; RRID: AB_1727493	1:400
CD11b-FITC; clone M1/70	BD Biosciences	Cat#: 557396; RRID: AB_396679	1:400
Ly-6A/E-FITC; clone D7	BD Biosciences	Cat#: 562058; RRID: AB_396688	1:400
Ter-119-Alexa Flour 700; clone TER-119	Biolegend	Cat#: 116220; RRID: AB_528963	1:400
Ly-6G/Ly-6C-Alexa Flour 700; clone RB6-8C5	Biolegend	Cat#: 108421; RRID: AB_493728	1:400
NK-1.1-Alexa Flour 700; clone PK136	Biolegend	Cat#: 108729; RRID: AB_2074426	1:400
CD3-Alexa Flour 700; clone 17A2	Biolegend	Cat#: 100215; RRID: AB_493696	1:400

CD45R/B220- Alexa Flour 700; clone RA3-6B2	Biolegend	Cat#: 103231; RRID: AB_493716	1:400
CD117 PE; clone 2B8	Biolegend	Cat#: 105808; RRID: AB_313217	1:400
CD117 PE594; clone 2B8	Biolegend	Cat#: 105834; RRID: AB_2564055	1:400
CD201-APC-eFlour780; clone eBio1560	Invitrogen	Cat#:47-2012-82; RRID: AB_2811822	1:400
DLL4-APC; clone HMD4-1	Biolegend	Cat#: 130814; RRID: AB_2092982	1:400

Table S3. Primers used for qRT-PCR

Primers for qRT-PCR	Sequences (5’-3’)
Atg5-Forward	TGTGCTTCGAGATGTGGTT
Atg5-Reverse	GTCAAATAGCTGACTCTTGGCAA
NCL-Forward	AAAGGCAAAAAGGCTACCACA
NCL-Reverse	GGAATGACTTTGGCTGGTGTAA
PTN-Forward	ATGTCGTCCCAGCAATATCAGC
PTN-Reverse	CCAAGATGAAAATCAATGCCAGG
MDK-Forward	GAAGAAGGCGCGGTACAATG
MDK-Reverse	GAGGTGCAGGGCTTAGTCA
