# Supplementary material for: Autophagy regulates the maturation of hematopoietic precursors in the embryo
Source: Nat Commun. 2024 Mar 15;15:2255. doi: 10.1038/s41467-024-46453-y (PMC10943005; doi:10.1038/s41467-024-46453-y)
Supplement: Supplementary file 3 — Description of Additional Supplementary Files [file 41467_2024_46453_MOESM3_ESM.pdf]

Description of Additional Supplementary Files:

Supplementary Data 1: Differential expression genes in the dis(nct clusters.

Supplementary Data 2: Gene list by Gene ontology biological processes(GOBP) and Kyoto Encyclopedia of Genes(KEGG) enrichment analysis.
