# Supplementary material for: Autophagy regulates the maturation of hematopoietic precursors in the embryo
Source: Nat Commun. 2024 Mar 15;15:2255. doi: 10.1038/s41467-024-46453-y (PMC10943005; doi:10.1038/s41467-024-46453-y)
Supplement: Supplementary file 6 — Reporting Summary [file 41467_2024_46453_MOESM6_ESM.pdf]

nature portfolio

Corresponding author(s): Zhuan Li

Last updated by author(s): Feb 12, 2024

Reporting Summary

Nature Portfolio wishes to improve the reproducibility of the work that we publish. This form provides structure for consistency and transparency in reporting. For further information on Nature Portfolio policies, see our Editorial Policies and the Editorial Policy Checklist.

Statistics

For all statistical analyses, confirm that the following items are present in the figure legend, table legend, main text, or Methods section.

n/a	Confirmed	
☐	☒	The exact sample size (*n*) for each experimental group/condition, given as a discrete number and unit of measurement
☐	☒	A statement on whether measurements were taken from distinct samples or whether the same sample was measured repeatedly
☐	☒	The statistical test(s) used AND whether they are one- or two-sided<br>*Only common tests should be described solely by name; describe more complex techniques in the Methods section.*
☒	☐	A description of all covariates tested
☐	☒	A description of any assumptions or corrections, such as tests of normality and adjustment for multiple comparisons
☐	☒	A full description of the statistical parameters including central tendency (e.g. means) or other basic estimates (e.g. regression coefficient) AND variation (e.g. standard deviation) or associated estimates of uncertainty (e.g. confidence intervals)
☐	☒	For null hypothesis testing, the test statistic (e.g. *F*, *t*, *r*) with confidence intervals, effect sizes, degrees of freedom and *P* value noted<br>*Give P values as exact values whenever suitable.*
☒	☐	For Bayesian analysis, information on the choice of priors and Markov chain Monte Carlo settings
☒	☐	For hierarchical and complex designs, identification of the appropriate level for tests and full reporting of outcomes
☒	☐	Estimates of effect sizes (e.g. Cohen's *d*, Pearson's *r*), indicating how they were calculated

Our web collection on statistics for biologists contains articles on many of the points above.

Software and code

Policy information about availability of computer code

Data collection	Seq libraries were produced with a Chromium system (10×Genomics, PN1000268) following the manufacture's instruction and sequenced on Illumina Novaseq 6000 platform in 150 bp paired-end manner (sequenced by Novogene and Berry Genomics).
Data analysis	Sequencing data from 10x Genomics was processed with the Cell Ranger software for each sample. Raw data in FASTQ format was processed and aligned to the mm10 mouse reference genome (https://cf.10xgenomics.com/supp/cell-exp/refdata-gex-mm10-2020-A.tar.gz) with the Cell Ranger v5.0.1 pipeline(10x Genomics, https://www.10xgenomics.com/). Doublet cells were removed using DoubletFinder R package (v2.0.3)71 with the recommended doublet rate by the pipeline. R package Seurat (v4.1.0)72 was used for downstream analysis and visualization. Raw counts were normalized with the "NormalizeData" function. Highly variable genes (HVGs) were selected by using the "FindVariableFeatures" function with default parameters, which were applied for principal-component analysis (PCA), remove batch effects, data integration and cell clustering. FACS data were analyzed using FlowJo (v10) .GraphPad (v8.0.2) was used for some statistical analysis. Immunofluorescence images were processed by Zeiss LSM 880 (blue) and image J.

For manuscripts utilizing custom algorithms or software that are central to the research but not yet described in published literature, software must be made available to editors and reviewers. We strongly encourage code deposition in a community repository (e.g. GitHub). See the Nature Portfolio guidelines for submitting code & software for further information.

Data

Policy information about availability of data

All manuscripts must include a data availability statement. This statement should provide the following information, where applicable:

- Accession codes, unique identifiers, or web links for publicly available datasets
- A description of any restrictions on data availability
- For clinical datasets or third party data, please ensure that the statement adheres to our policy

The raw sequencing data generated in the present study are deposited in the GSA (Genome Sequence Archive in BIG Data Center, Beijing Institute of Genomics, Chinese Academy of Sciences) with the accession number (CRA008812 https://ngdc.cncb.ac.cn/gsa/s/7ep9swyS). The processed datasets generated this study are deposited in the OMIX China National Center for Bioinformation/Beijing Institute of Genomics, Chinese Academy of Sciences), under accession (OMIX002331 https://ngdc.cncb.ac.cn/omix/view/OMIX002331).

Research involving human participants, their data, or biological material

Policy information about studies with human participants or human data. See also policy information about sex, gender (identity/presentation), and sexual orientation and race, ethnicity and racism.

Reporting on sex and gender	N/A
Reporting on race, ethnicity, or other socially relevant groupings	N/A
Population characteristics	N/A
Recruitment	N/A
Ethics oversight	N/A

Note that full information on the approval of the study protocol must also be provided in the manuscript.

Field-specific reporting

Please select the one below that is the best fit for your research. If you are not sure, read the appropriate sections before making your selection.

[x] Life sciences [ ] Behavioural & social sciences [ ] Ecological, evolutionary & environmental sciences

For a reference copy of the document with all sections, see nature.com/documents/nr-reporting-summary-flat.pdf

Life sciences study design

All studies must disclose on these points even when the disclosure is negative.

Sample size	Sample size was determined based on consensus practice in the field.<br>Basically, for flow cytometric analysis a total of n= 3 or more embryos were analyzed with consistent results. For cell staining, at least 15 cells were unbiased imaged from 3 or more independent experiments (because of rare cell number, flow cytometry sorting was performed from at least 3 pooled embryos). For qPCR analysis, three or more independent experiments were performed (pooled cDNA extracted from 3 or more embryos). For 10x genomics samples, three independent experiments were performed from 6 control or ATG5 knockout embryos.
Data exclusions	There was no data excluded from the analysis.
Replication	Majority of experiments were repeated at least three times to ensure reproducibility. Number of replicates in indicated in the figure legends. All attempts to replicate experiments were successful.
Randomization	Randomization was performed.
Blinding	Experimental analysis was performed blinded for embryos genetic experiments. In some cases, the embryos were genotyped after the experimental procedure was performed. In other cases, control and experimental conditions were labeled with numbers by other lab mates to ensure single-blind study.

Behavioural & social sciences study design

All studies must disclose on these points even when the disclosure is negative.

Study description	*Briefly describe the study type including whether data are quantitative, qualitative, or mixed-methods (e.g. qualitative cross-sectional, quantitative experimental, mixed-methods case study).*
Research sample	*State the research sample (e.g. Harvard university undergraduates, villagers in rural India) and provide relevant demographic information (e.g. age, sex) and indicate whether the sample is representative. Provide a rationale for the study sample chosen. For studies involving existing datasets, please describe the dataset and source.*
Sampling strategy	*Describe the sampling procedure (e.g. random, snowball, stratified, convenience). Describe the statistical methods that were used to predetermine sample size OR if no sample-size calculation was performed, describe how sample sizes were chosen and provide a rationale for why these sample sizes are sufficient. For qualitative data, please indicate whether data saturation was considered, and what criteria were used to decide that no further sampling was needed.*
Data collection	*Provide details about the data collection procedure, including the instruments or devices used to record the data (e.g. pen and paper, computer, eye tracker, video or audio equipment) whether anyone was present besides the participant(s) and the researcher, and whether the researcher was blind to experimental condition and/or the study hypothesis during data collection.*
Timing	*Indicate the start and stop dates of data collection. If there is a gap between collection periods, state the dates for each sample cohort.*
Data exclusions	*If no data were excluded from the analyses, state so OR if data were excluded, provide the exact number of exclusions and the rationale behind them, indicating whether exclusion criteria were pre-established.*
Non-participation	*State how many participants dropped out/declined participation and the reason(s) given OR provide response rate OR state that no participants dropped out/declined participation.*
Randomization	*If participants were not allocated into experimental groups, state so OR describe how participants were allocated to groups, and if allocation was not random, describe how covariates were controlled.*

Ecological, evolutionary & environmental sciences study design

All studies must disclose on these points even when the disclosure is negative.

Study description	*Briefly describe the study. For quantitative data include treatment factors and interactions, design structure (e.g. factorial, nested, hierarchical), nature and number of experimental units and replicates.*
Research sample	*Describe the research sample (e.g. a group of tagged Passer domesticus, all Stenocereus thurberi within Organ Pipe Cactus National Monument), and provide a rationale for the sample choice. When relevant, describe the organism taxa, source, sex, age range and any manipulations. State what population the sample is meant to represent when applicable. For studies involving existing datasets, describe the data and its source.*
Sampling strategy	*Note the sampling procedure. Describe the statistical methods that were used to predetermine sample size OR if no sample-size calculation was performed, describe how sample sizes were chosen and provide a rationale for why these sample sizes are sufficient.*
Data collection	*Describe the data collection procedure, including who recorded the data and how.*
Timing and spatial scale	*Indicate the start and stop dates of data collection, noting the frequency and periodicity of sampling and providing a rationale for these choices. If there is a gap between collection periods, state the dates for each sample cohort. Specify the spatial scale from which the data are taken*
Data exclusions	*If no data were excluded from the analyses, state so OR if data were excluded, describe the exclusions and the rationale behind them, indicating whether exclusion criteria were pre-established.*
Reproducibility	*Describe the measures taken to verify the reproducibility of experimental findings. For each experiment, note whether any attempts to repeat the experiment failed OR state that all attempts to repeat the experiment were successful.*
Randomization	*Describe how samples/organisms/participants were allocated into groups. If allocation was not random, describe how covariates were controlled. If this is not relevant to your study, explain why.*
Blinding	*Describe the extent of blinding used during data acquisition and analysis. If blinding was not possible, describe why OR explain why blinding was not relevant to your study.*

Did the study involve field work? ☐ Yes ☐ No

Field work, collection and transport

Field conditions	*Describe the study conditions for field work, providing relevant parameters (e.g. temperature, rainfall).*
Location	*State the location of the sampling or experiment, providing relevant parameters (e.g. latitude and longitude, elevation, water depth).*
Access & import/export	*Describe the efforts you have made to access habitats and to collect and import/export your samples in a responsible manner and in compliance with local, national and international laws, noting any permits that were obtained (give the name of the issuing authority, the date of issue, and any identifying information).*
Disturbance	*Describe any disturbance caused by the study and how it was minimized.*

Reporting for specific materials, systems and methods

We require information from authors about some types of materials, experimental systems and methods used in many studies. Here, indicate whether each material, system or method listed is relevant to your study. If you are not sure if a list item applies to your research, read the appropriate section before selecting a response.

Materials & experimental systems

n/a	Involved in the study	
☐	☒	Antibodies
☐	☒	Eukaryotic cell lines
☒	☐	Palaeontology and archaeology
☐	☒	Animals and other organisms
☒	☐	Clinical data
☒	☐	Dual use research of concern
☒	☐	Plants

Methods

n/a	Involved in the study	
☒	☐	ChIP-seq
☐	☒	Flow cytometry
☒	☐	MRI-based neuroimaging

Antibodies

Antibodies used

The following antibodies were used for immunostaining: CD34 Monclonal antibody; clone RAM34 from eBioscience (1:200,Cat#:13-0341-82; RRID: AB_466425), CD117 clone 2B8 from BD Biosciences (1:100,Cat#: 553352; RRID: AB_394803), Runx1 antibody [EPR3099] from Abcam (1:500,Cat# ab92336;RRID:AB_2049267), Nucleolin (D4C7O) from Cell Signalling Technolog (1:100,Cat#: 14574), Anti-SQSTM1 / p62 antibody [EPR4844] from Abcam (1:400,Cat# ab109012), Anti-LAMP1 antibody [EPR21026] from Abcam (1:100,Cat# ab208943), Anti-GFP (Green Fluorescent Protein) pAb from MBL (1:200,Cat# 598), Anti-RFP (GOAT) Antibody from Rockland (1:200,Cat# 200-101-379), ABflo® 750-conjugated Goat Anti-Rabbit from Abclonal(1:1000,Cat#: AS072; RRID: AB_2768332), Alexa Flour 488 goat anti-rabbit IgG(H+L), Alexa Flour 647 goat anti-rat IgG, SA1010STREPTAVIDIN CY3 from Invitrogen (1:1000,Cat#: A11008; RRID: AB_143165;1:500,Cat#: A21247; RRID: AB_141778;1:1000,Cat#: SA1010).

The antibodies for FACS were diluted 1:400 in staining procedures.

The following FACS antibodies were purchased from BioLegend: CD31-PE [MEC13.3](Cat#:102508:RRID:AB 312915),CD31-APC [MEC13.3] (Cat#:102510:RRID:AB 312917),CD31-Alexa Flour 700[MEC13.3](Cat#:102444;RRID:AB 2832289),D41-BV510[MWReg30](Cat#:133923:RRID:AB 2564013),CD41-PE-Cyanine7[MWReg30](Cat#: 133916;RRID:AB 11124102),CD45-APC [clone 30-F11](Cat#: 103112; RRID: AB_312977),CD44-APC-Cy7 [clone IM7](Cat#: 103028; RRID: AB_830785), Ter-119-Alexa Flour700[clone TER-119] (Cat#:116220.RRID:AB 528963),Ly-6G/Ly-6C-Alexa Flour 700[clone RB6-8C5](Cat#:108421; RRID:AB 493728),NK-1.1-Alexa Flour 700 [clone PK136](Cat#:108729,RRID:AB 2074426),CD3-Alexa Flour 700[clone 17A2](Cat#:100215RRID:AB 493696),CD45R/B220- Alexa Flour 700[clone RA3-6B2](Ca#: 103231; RRID:AB 493716),CD117 PE[clone 2B8](Cat#: 105808; RRID: AB_313217),CD117 PE594 [clone 2B8](Cat#: 105834; RRID: AB_2564055),DLL4-APC[clone HMD4-1](Cat#: 130814; RRID: AB_2092982)The following FACS antibodies were purchased from BD Biosciences:CD41-FITC[MWReg30](Cat#: 553848:RRID:AB 395085),CD45-PE [clone 30-F11] (Cat#: 553081; RRID: AB_394611),CD43-BV421 [clone S7](Cat#: 562958; RRID: AB_2665409),CD45-FITC [clone 30-F11](Cat#: 553079; RRID: AB_394609),CD44-BV605 [clone IM7](Cat#: 563058; RRID: AB_2737979),CD45.1-APC [clone A20](Cat#: 558701; RRID: AB_1645214),CD45.2-PE [clone 104](Cat#: 560695; RRID: AB_1727493),CD11b-FITC [clone M1/70](Cat#: 557396; RRID: AB_396679),Ly-6A/E-FITC [clone D7](Cat#: 562058; RRID: AB_396688).CD201-APC-eFlour780[clone eBio1560] from Invitrogen (Cat#:47-2012-82; RRID: AB_2811822).

Validation

Validation performed by the company for these clones biolegend "This product lot has passed BioLegend's QC testing and is certified for use", for Thermofisher "Advanced Verification: This Antibody was verified by Cell treatment to ensure that the antibody binds to the antigen stated.", for BD pharmingen "This product complies with all BDB release criteria". Following commercial antibodies were validated by the manufacturers as indicated on their websites:

CD34 Monclonal antibody (https://www.thermofisher.cn/cn/zh/antibody/product/CD34-Antibody-clone-RAM34-Monoclonal/13-0341-82)

Runx1 antibody [EPR3099l from Abcam (https://www.abcam.cn/products/primary-antibodies/runx1--aml1--runx3--runx2-antibody-epr3099-ab92336.html)

Nucleolin from cell signaling Technology (https://www.cellsignal.cn/products/primary-antibodies/nucleolin-d4c7o-rabbit-mab/14574)

AntiSOSTM1 / p62 antibody [EPR4844] from Abcam (https://www.abcam.cn/products/primary-antibodies/sqstm1--p62-antibody-

	epr4844-autophagosome-marker-ab109012.html)<br>Ant-LAMP1 antibody [EPR21026] from Abcam (https://www.abcam.cn/products/primary-antibodies/lamp1-antibody-epr21026-ab208943.html)<br>Anti-GFP (Green Fluorescent Protein) pAb from MBL (https://www.mblbio.com/bio/g/dtl/A/?pcd=598)<br>Ant-RFP (GOAT) Antibody from Rockland (https://www.rockland.com/categories/primary-antibodies/rfp-antibody-200-101-379/)

Eukaryotic cell lines

Policy information about cell lines and Sex and Gender in Research

Cell line source(s)	OP9-DL1 cell line is gift from Elaine Dzierzak. OP9 cell line is gift from Yu Zhang.
Authentication	No authentication was performed.
Mycoplasma contamination	OP9-DL1 cell line and OP9 cell line tested negative for mycoplasma.
Commonly misidentified lines<br>(See ICLAC register)	None of the cell lines used are listed as commonly misidentified cell lines in the ICLAC register.

Palaeontology and Archaeology

Specimen provenance	*Provide provenance information for specimens and describe permits that were obtained for the work (including the name of the issuing authority, the date of issue, and any identifying information). Permits should encompass collection and, where applicable, export.*
Specimen deposition	*Indicate where the specimens have been deposited to permit free access by other researchers.*
Dating methods	*If new dates are provided, describe how they were obtained (e.g. collection, storage, sample pretreatment and measurement), where they were obtained (i.e. lab name), the calibration program and the protocol for quality assurance OR state that no new dates are provided.*

☐ Tick this box to confirm that the raw and calibrated dates are available in the paper or in Supplementary Information.

Ethics oversight	*Identify the organization(s) that approved or provided guidance on the study protocol, OR state that no ethical approval or guidance was required and explain why not.*

Note that full information on the approval of the study protocol must also be provided in the manuscript.

Animals and other research organisms

Policy information about studies involving animals; ARRIVE guidelines recommended for reporting animal research, and Sex and Gender in Research

Laboratory animals	The following mouse strain were used in this study: C57BL/6]-Ly5.2/2, C57BL/6J-Ly5.1/1, LC3-RFP-EGFP, ATG5f/f, VE-cadherin-Cre. Males and females of age 8-20 weeks were used in timing mating. Embryos were harvested at E9-12.5 for experiments. C57BL/6-Ly5.1/161 (8-12 weeks) mice were as transplantation recipients. All mice were horsed in SPF conditions with 12 light/12 dark cycle, temperatures of 20-26°C and humidity of 40-60%.
Wild animals	No wild animals have been used in the study.
Reporting on sex	This information has not been collected
Field-collected samples	This study did not involve samples collected from the field.
Ethics oversight	All animal protocols were approved by the ethics committee of Southern Medical University.

Note that full information on the approval of the study protocol must also be provided in the manuscript.

Clinical data

Policy information about clinical studies

All manuscripts should comply with the ICMJEguidelines for publication of clinical research and a completedCONSORT checklist must be included with all submissions.

Clinical trial registration	*Provide the trial registration number from ClinicalTrials.gov or an equivalent agency.*
Study protocol	*Note where the full trial protocol can be accessed OR if not available, explain why.*
Data collection	*Describe the settings and locales of data collection, noting the time periods of recruitment and data collection.*

Outcomes | *Describe how you pre-defined primary and secondary outcome measures and how you assessed these measures.*

Dual use research of concern

Policy information about [dual use research of concern]

Hazards

Could the accidental, deliberate or reckless misuse of agents or technologies generated in the work, or the application of information presented in the manuscript, pose a threat to:

No	Yes	
☐	☐	Public health
☐	☐	National security
☐	☐	Crops and/or livestock
☐	☐	Ecosystems
☐	☐	Any other significant area

Experiments of concern

Does the work involve any of these experiments of concern:

No	Yes	
☐	☐	Demonstrate how to render a vaccine ineffective
☐	☐	Confer resistance to therapeutically useful antibiotics or antiviral agents
☐	☐	Enhance the virulence of a pathogen or render a nonpathogen virulent
☐	☐	Increase transmissibility of a pathogen
☐	☐	Alter the host range of a pathogen
☐	☐	Enable evasion of diagnostic/detection modalities
☐	☐	Enable the weaponization of a biological agent or toxin
☐	☐	Any other potentially harmful combination of experiments and agents

Plants

Seed stocks | *Report on the source of all seed stocks or other plant material used. If applicable, state the seed stock centre and catalogue number. If plant specimens were collected from the field, describe the collection location, date and sampling procedures.*

Novel plant genotypes | *Describe the methods by which all novel plant genotypes were produced. This includes those generated by transgenic approaches, gene editing, chemical/radiation-based mutagenesis and hybridization. For transgenic lines, describe the transformation method, the number of independent lines analyzed and the generation upon which experiments were performed. For gene-edited lines, describe the editor used, the endogenous sequence targeted for editing, the targeting guide RNA sequence (if applicable) and how the editor was applied.*

Authentication | *Describe any authentication procedures for each seed stock used or novel genotype generated. Describe any experiments used to assess the effect of a mutation and, where applicable, how potential secondary effects (e.g. second site T-DNA insertions, mosiacism, off-target gene editing) were examined.*

ChIP-seq

Data deposition

☐ Confirm that both raw and final processed data have been deposited in a public database such as [GEO].

☐ Confirm that you have deposited or provided access to graph files (e.g. BED files) for the called peaks.

Data access links
May remain private before publication. | *For "Initial submission" or "Revised version" documents, provide reviewer access links. For your "Final submission" document, provide a link to the deposited data.*

Files in database submission | *Provide a list of all files available in the database submission.*

Genome browser session
(e.g. [UCSC]) | *Provide a link to an anonymized genome browser session for "Initial submission" and "Revised version" documents only, to enable peer review. Write "no longer applicable" for "Final submission" documents.*

Methodology

Replicates	*Describe the experimental replicates, specifying number, type and replicate agreement.*
Sequencing depth	*Describe the sequencing depth for each experiment, providing the total number of reads, uniquely mapped reads, length of reads and whether they were paired- or single-end.*
Antibodies	*Describe the antibodies used for the ChIP-seq experiments; as applicable, provide supplier name, catalog number, clone name, and lot number.*
Peak calling parameters	*Specify the command line program and parameters used for read mapping and peak calling, including the ChIP, control and index files used.*
Data quality	*Describe the methods used to ensure data quality in full detail, including how many peaks are at FDR 5% and above 5-fold enrichment.*
Software	*Describe the software used to collect and analyze the ChIP-seq data. For custom code that has been deposited into a community repository, provide accession details.*

Flow Cytometry

Plots

Confirm that:

- [x] The axis labels state the marker and fluorochrome used (e.g. CD4-FITC).
- [x] The axis scales are clearly visible. Include numbers along axes only for bottom left plot of group (a 'group' is an analysis of identical markers).
- [x] All plots are contour plots with outliers or pseudocolor plots.
- [x] A numerical value for number of cells or percentage (with statistics) is provided.

Methodology

Sample preparation	AGM were dissected from mouse embryos and dissociated in PBS with 10% fetal bovine serum and 0.125% collagenase I. Digested cells were stained by antibody combination: CD31, CD41, CD45, CD43, CD44, DLL4, CD201, cKit for HECs and pre-HSCs. For cell cycle, Ki67 and Dapi were used. 7AAD or Hoechst was used for dead cell exclusion.
Instrument	Cells were sorted on a BD FACSAria lll or BD FACSAria Fusion instrument.
Software	Flow cytometry data was collected by using BD FACSDiva Software and analyzed by using FlowJo v10.
Cell population abundance	Cell purity post-sort was not assessed given the very small number of cells collected per sample.
Gating strategy	For all FACS experiments, the cell population was defined based on FSC-A and SSC-A parameters to remove debris. Doublets were removed based on FSC-A and FSC-H parameters and viable single cells were determined by 7AAD or Hoechst staining. Gating for fluorochromes was defined based on negative controls.

- [x] Tick this box to confirm that a figure exemplifying the gating strategy is provided in the Supplementary Information.

Magnetic resonance imaging

Experimental design

Design type	*Indicate task or resting state; event-related or block design.*
Design specifications	*Specify the number of blocks, trials or experimental units per session and/or subject, and specify the length of each trial or block (if trials are blocked) and interval between trials.*
Behavioral performance measures	*State number and/or type of variables recorded (e.g. correct button press, response time) and what statistics were used to establish that the subjects were performing the task as expected (e.g. mean, range, and/or standard deviation across subjects).*

Acquisition

Imaging type(s) — *Specify: functional, structural, diffusion, perfusion.*

Field strength — *Specify in Tesla*

Sequence & imaging parameters — *Specify the pulse sequence type (gradient echo, spin echo, etc.), imaging type (EPI, spiral, etc.), field of view, matrix size, slice thickness, orientation and TE/TR/flip angle.*

Area of acquisition — *State whether a whole brain scan was used OR define the area of acquisition, describing how the region was determined.*

Diffusion MRI ☐ Used ☐ Not used

Preprocessing

Preprocessing software — *Provide detail on software version and revision number and on specific parameters (model/functions, brain extraction, segmentation, smoothing kernel size, etc.).*

Normalization — *If data were normalized/standardized, describe the approach(es): specify linear or non-linear and define image types used for transformation OR indicate that data were not normalized and explain rationale for lack of normalization.*

Normalization template — *Describe the template used for normalization/transformation, specifying subject space or group standardized space (e.g. original Talairach, MNI305, ICBM152) OR indicate that the data were not normalized.*

Noise and artifact removal — *Describe your procedure(s) for artifact and structured noise removal, specifying motion parameters, tissue signals and physiological signals (heart rate, respiration).*

Volume censoring — *Define your software and/or method and criteria for volume censoring, and state the extent of such censoring.*

Statistical modeling & inference

Model type and settings — *Specify type (mass univariate, multivariate, RSA, predictive, etc.) and describe essential details of the model at the first and second levels (e.g. fixed, random or mixed effects; drift or auto-correlation).*

Effect(s) tested — *Define precise effect in terms of the task or stimulus conditions instead of psychological concepts and indicate whether ANOVA or factorial designs were used.*

Specify type of analysis: ☐ Whole brain ☐ ROI-based ☐ Both

Statistic type for inference — *Specify voxel-wise or cluster-wise and report all relevant parameters for cluster-wise methods.*

(See Eklund et al. 2016)

Correction — *Describe the type of correction and how it is obtained for multiple comparisons (e.g. FWE, FDR, permutation or Monte Carlo).*

Models & analysis

n/a	Involved in the study
☐	☐ Functional and/or effective connectivity
☐	☐ Graph analysis
☐	☐ Multivariate modeling or predictive analysis

Functional and/or effective connectivity — *Report the measures of dependence used and the model details (e.g. Pearson correlation, partial correlation, mutual information).*

Graph analysis — *Report the dependent variable and connectivity measure, specifying weighted graph or binarized graph, subject- or group-level, and the global and/or node summaries used (e.g. clustering coefficient, efficiency, etc.).*

Multivariate modeling and predictive analysis — *Specify independent variables, features extraction and dimension reduction, model, training and evaluation metrics.*
